# Association between hypothermic machine perfusion parameters and graft function in deceased donor kidney transplantation

**DOI:** 10.1080/07853890.2026.2634488

**Published:** 2026-02-25

**Authors:** Boqing Dong, Yuting Zhao, Yang Li, Huanjing Bi, Chongfeng Wang, Ying Wang, Jingwen Wang, Zuhan Chen, Cuinan Lu, Xiaoming Ding

**Affiliations:** a Department of Renal Transplantation, The First Affiliated Hospital of Xi’an Jiaotong University, Xi’an, China; b Department of Gynecologic Oncology, National Cancer Center, National Clinical Research Center for Cancer, Cancer Hospital, Chinese Academy of Medical Sciences, Peking Union Medical College, Beijing, China; c Chinese Academy of Medical Sciences, Peking Union Medical College, Beijing, China

**Keywords:** Kidney transplantation, delayed graft function, hypothermic machine perfusion, non-linear relationships, machine learning

## Abstract

**Background:**

Kidney transplantation (KT) is the most effective treatment for end-stage renal disease. Hypothermic machine perfusion (HMP) can improve renal energy metabolism and reduce ischemia-reperfusion injury compared with static cold storage. This study aimed to evaluate the association between HMP parameters and graft function in deceased donor kidney transplantation (DDKT) and to develop a predictive model for early risk stratification.

**Methods:**

A retrospective analysis was conducted on 2,041 DDKT recipients from 1 January 2015 to 30 June 2023. The primary outcome, delayed graft function (DGF), was defined as the need for at least one dialysis session within the first week after transplantation. Consensus clustering (CC) and restricted cubic spline (RCS) analysis were used to evaluate the associations between clinical data, HMP parameters, and graft function. Feature selection was performed using Lasso-penalized logistic regression (LR), and multivariable LR was used to construct the predictive model. The model’s performance was assessed using the area under the curve (AUC), calibration curves, and decision curve analysis (DCA).

**Results:**

Among the DDKT recipients, 12.9% developed DGF. HMP parameters varied significantly between the two groups, with DGF recipients showing distinct patterns in perfusion resistance, flux, and pressure. CC identified two recipient clusters with distinct DGF risk profiles, graft function, and donor characteristics. Non-linear relationships were identified between HMP parameters and DGF risk, with thresholds for initial resistance, terminal resistance, and terminal flux. The predictive model integrating six variables achieved an AUC of 0.78 (95% CI: 0.76–0.82) in the test set. Calibration and DCA confirmed good reliability and net clinical benefit.

**Conclusion:**

Non-linear relationships between HMP parameters and DGF underscore graft perfusion complexity. The proposed model demonstrated robust internal performance and may support early post-transplant risk stratification. External validation in independent cohorts is warranted to confirm generalizability and clinical applicability.

## 1. Introduction

Chronic kidney disease (CKD) is a major global health burden, affecting over 10% of the world’s population [1,2]. For patients with end-stage renal disease (ESRD), kidney transplantation (KT) is the preferred treatment, offering superior long-term survival and quality of life compared to dialysis [3,4]. However, the success of KT is closely linked to graft preservation strategies, particularly in the context of deceased donor kidney transplantation (DDKT), where ischemia-reperfusion injury (IRI) represents a key determinant of post-transplantation outcomes [5–7].

Hypothermic machine perfusion (HMP) has emerged as an organ preservation technique designed to mitigate IRI by providing continuous cold perfusion with preservation solutions [8,9]. Unlike static cold storage (SCS), which relies on passive cooling and is associated with significant metabolic suppression but limited tissue protection, HMP offers dynamic perfusion, facilitating the removal of metabolic waste, reducing oxidative stress, and improving microvascular integrity [10,11]. Additionally, HMP allows for real-time monitoring of perfusion parameters, such as flux, resistance, and pressure, providing valuable insights into graft viability and potential function post-transplantation [12].

Despite these advantages, the precise relationship between HMP parameters and graft function has not been fully elucidated. In particular, delayed graft function (DGF) represents a complex and multifactorial early outcome influenced by donor, recipient, and perioperative factors. Although DGF primarily affects early graft recovery and length of hospitalization, it also serves as a clinically relevant indicator of perioperative graft injury and has been associated with an increased risk of acute rejection (AR) and inferior long-term graft outcomes in selected settings [13,14]. From a clinical perspective, early identification of recipients at high risk of DGF may enable individualized perioperative management and closer post-transplant monitoring, rather than informing decisions regarding organ acceptance or waiting-list allocation.

In the present study, we conducted a retrospective analysis of DDKT recipients whose donor kidneys were preserved using HMP at our center. We aimed to systematically evaluate the association between HMP parameters and early graft function, including DGF occurrence and post-transplant serum creatinine (Scr) trajectories. Furthermore, we sought to develop an internally validated prediction model to support early post-transplant risk stratification within this specific clinical setting, while providing a structured framework for future external validation and biomarker integration.

## 2. Materials and methods

### 2.1. Study cohort and ethics approval

Data on donors and recipients at our institution from 1 January 2015 to 30 June 2023 were extracted from the electronic medical record system. All kidneys in this study were legally obtained from deceased donors (DD), including donation after circulatory death (DCD) and donation after brain death. Donor organs were procured by the organ procurement organization of our center and allocated through the China Organ Transplant Response System.

All recipients underwent intravenous induction therapy with either rabbit anti-thymocyte globulin (5 mg/kg, administered over four daily doses), basiliximab (20 mg on post-transplant days 0 and 4), or alemtuzumab (30 mg as a single dose or 15 mg on post-transplant days 0 and 1), depending on recipient characteristics. Maintenance immunosuppression consisted of a triple-drug regimen, including mycophenolic acid, a calcineurin inhibitor (CNI), and prednisone. This retrospective, non-interventional study complied with the principles of the Declaration of Helsinki and was approved by the Ethics Committee of the First Affiliated Hospital of Xi’an Jiaotong University (Approval No. XJTU1AF2023LSK-450). De-identified clinical data were used to protect patient privacy and confidentiality. Given the retrospective design and the use of anonymized data, the requirement for informed consent was formally waived by the Ethics Committee of the First Affiliated Hospital of Xi’an Jiaotong University.

### 2.2. Inclusion and exclusion criteria

**Figure 1.**
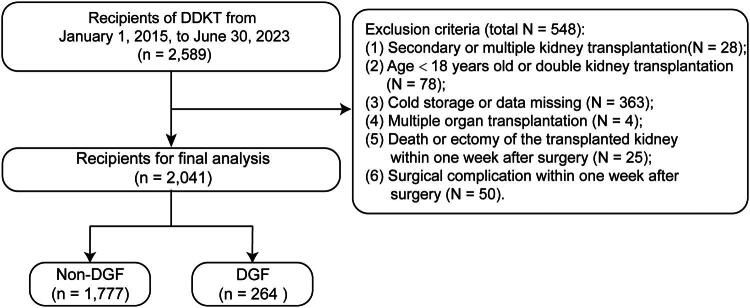
The flowchart of recipients’ selection in this study. DDKT: deceased donor kidney transplantation; HMP: hypothermic machine perfusion; DGF: delayed graft function.

The study flowchart is presented in Figure 1. This study included recipients undergoing their first DDKT at our institution who were aged 18 years or older and received a single kidney transplant. Recipients were excluded if they had previously undergone multiple KTs, received bilateral KTs, received kidneys preserved *via* SCS, or had incomplete data. Additionally, recipients were excluded if they died, underwent graft nephrectomy, or developed surgical complications within the first week post-transplantation.

### 2.3. The definition of outcomes and selected variables

The primary outcome, DGF, was defined as the requirement for at least one dialysis session within the first week post-transplantation [14]. Furthermore, Scr levels within one year were considered the secondary outcome. Donor clinical data included kidney laterality, presence of polar artery, sex, body mass index (BMI), age, primary disease, history of hypertension, cold ischemia time (CIT), and warm ischemia time (WIT). Donor laboratory tests were performed before organ procurement and included alanine aminotransferase (ALT), aspartate aminotransferase (AST), total bilirubin (Tbil), direct Bilirubin (Dbil), total protein (TP), albumin (ALB), gamma-glutamyl transferase (GGT), Scr, blood urea nitrogen (BUN), uric acid (UA), urinary protein, hemoglobin (Hb), platelets (PLT), white blood cell (WBC), potassium, sodium, and calcium. Recipient data collected included sex, age, number of human leukocyte antigen mismatches, BMI, and panel-reactive antibodies.

All donor kidneys were routinely placed on the LifePort (Organ Recovery Systems, Chicago, IL, USA) immediately after procurement and maintained to continuous cold perfusion. In our study, the perfusion protocol was standardized: University of Wisconsin (UW) solution was used for both *in situ* and initial ex situ flushing, followed by the KPS-1 solution during non-oxygenated HMP. Initial parameters following machine setup and terminal parameters before surgery were recorded, including flux, pressure, and resistance. Additionally, kidney perfusion time was documented and included in the analysis. When a polar artery was present, it was routinely reconstructed with the main renal artery before connection to the LifePort, ensuring full perfusion of the graft. All kidneys were connected to the device using a Sealring cannula (CAN0720), which preserves the arterial valve and provides a secure fixation.

### 2.4. Statistical analyses

Continuous variables were expressed as medians with interquartile ranges and compared using the Kruskal-Wallis test. Categorical variables were summarized as frequencies (percentages) and compared using the Chi-square test. For multi-categorical variables, we additionally performed Fisher’s exact test for each category *versus* all other categories, with *p*-values adjusted for multiple comparisons using the Benjamini–Hochberg method.

Univariate logistic regression (LR) analyses were performed as an initial exploratory screening step to identify candidate variables potentially associated with DGF. To account for multiple testing in these univariate analyses, *p*-values derived from univariate LR were adjusted using the Benjamini–Hochberg false discovery rate (FDR) method. Due to the non-normal distribution of biochemical test results and the tendency for odds ratios (ORs) to approach unity when modeled on the original scale, biochemical variables were log_2_-transformed before univariate LR analysis to better characterize their association with DGF. Log_2_-transformed biochemical parameters were also incorporated as candidate predictors in the modeling process.

Consensus clustering (CC), an unsupervised clustering method, was used to stratify recipients into distinct clusters, facilitating the identification of novel disease subtypes and enabling comparative analyses among groups [15]. To explore the association between HMP parameters and donor characteristics, recipients were clustered using the K-means algorithm based on HMP parameters. The optimal number of clusters was determined using consensus matrix heatmaps and consensus cumulative distribution function plots [16]. DGF risk, donor characteristics, HMP parameters, and graft function were then compared across clusters.

Restricted cubic spline (RCS) analysis was performed to investigate potential non-linear associations between HMP parameters and DGF. RCS is particularly suitable for modeling complex relationships while preserving the local structure of the data [17]. Models with five knots were used to evaluate the associations between HMP parameters and DGF risk.

The dataset was randomly split, allocating 70% to the train set and 30% to the test set. The train set was used for feature selection and predictive model development, while the test set was reserved for performance evaluation. To enhance feature selection and model robustness, Lasso-penalized LR was employed. Lasso, a regularization technique, shrinks regression coefficients and eliminates noncontributory variables by setting them to 0 [18]. Ten-fold cross-validation was employed to determine the optimal penalty level, minimizing overfitting within the train cohort. Subsequently, the multivariable LR model was used to develop the predictive model on the train set. To assess multicollinearity among these features, the variance inflation factor (VIF) was calculated, with VIF values <5 indicating no concerning multicollinearity, thereby ensuring model stability and interpretability.

Model performance was evaluated in terms of discrimination, calibration, and clinical utility. Discrimination was assessed using receiver operating characteristic (ROC) analysis, while calibration was evaluated in both the train and test sets using calibration curves. Decision curve analysis (DCA) was performed to assess clinical utility. Overall model fit was additionally quantified using Nagelkerke’s pseudo-*R*^2^, a commonly used measure for logistic regression models that reflects the improvement of the fitted model over the null model. Finally, a nomogram was constructed to represent the predictive model, and an interactive web-based version was also developed to facilitate convenient clinical application.

All statistical analyses were conducted in R (version 4.2.3). Details of the R packages and their respective versions are provided in Table S1. Statistical significance was set at a two-sided *p*-value <0.05.

## 3. Results

### 3.1. Baseline characteristics of donor and recipient

Of the 2,589 recipients who underwent DDKT at our institution, 548 were excluded based on predefined criteria (Figure 1). Ultimately, 2,041 recipients were included in the analysis, with 264 (12.9%) meeting the diagnostic criteria for DGF. The age of recipients ranged from 19 to 67 years, while donor age ranged from 6 to 75 years.

As shown in Table 1, statistically significant differences were identified in both donor and recipient characteristics between the DGF and non-DGF groups. Donors in the DGF group had significantly higher BMI, CIT, ALT, AST, Dbil, GGT, Scr, BUN, and UA levels, whereas TP, ALB, and PLT levels were significantly lower in the DGF group than in the non-DGF group (*p* < 0.05). Recipients whose donors had a history of hypertension, tested positive for urinary protein, or were donors from DCD were more likely to develop DGF. Furthermore, primary disease distribution differed significantly between the DGF and non-DGF groups (*p* < 0.05). Recipients of kidneys from donors whose primary disease was cerebrovascular disease were more likely to develop DGF (*p* < 0.05). In terms of recipient characteristics, the DGF group had a significantly higher proportion of males and a higher BMI (*p* < 0.05).

**Table 1.** Baseline characteristics of donors and recipients stratified by DGF status.

	Overall	Non-DGF	DGF	*p-*Value
*N*	2,041	1,777 (87.1%)	264 (12.9%)	
Donor variables				
Right donor kidney, *n* (%)	1,013 (49.6)	870 (49.0)	143 (54.2)	0.130
Polar artery, *n* (%)	385 (18.9)	329 (18.5)	56 (21.2)	0.337
Male donor, *n* (%)	1,630 (79.9)	1,420 (79.9)	210 (79.5)	0.956
Donor BMI (kg/m^2^, M [P25, P75])	22.60 [20.81, 24.49]	22.49 [20.76, 24.49]	22.99 [21.22, 25.18]	0.034
Donor age (years, M [P25, P75])	52.00 [43.00, 60.00]	52.00 [43.00, 60.00]	52.00 [43.75, 62.00]	0.325
Primary disease, *n* (%)				<0.001
Cerebrovascular disease	986 (48.3)	830 (46.7)	156 (59.1)	<0.001
Craniocerebral trauma	764 (37.4)	693 (39.0)	71 (26.9)	<0.001
Hypoxic ischemic encephalopathy	172 (8.4)	159 (8.9)	13 (4.9)	0.010
Tumor	103 (5.0)	80 (4.5)	23 (8.7)	0.710
Others	16 (0.8)	15 (0.8)	1 (0.4)	0.040
History of hypertension, *n* (%)	1,020 (50.0)	841 (47.3)	179 (67.8)	<0.001
CIT (h, M [P25, P75])	9.0 [6.0, 11.0]	9.0 [6.0, 11.0]	9.0 [7.0, 12.0]	0.006
WIT (min, M [P25, P75])	6 [4,8]	6 [3,8]	6 [4,8]	0.059
ALT (U/L, M [P25, P75])	37.00 [23.00, 73.00]	36.00 [23.00, 69.00]	42.00 [26.00, 104.75]	<0.001
AST (U/L, M [P25, P75])	47.30 [29.00, 92.00]	47.00 [29.00, 89.00]	56.00 [32.00, 142.75]	<0.001
Tbil (μmol/L, M [P25, P75])	15.70 [10.68, 23.50]	15.50 [10.70, 23.50]	16.35 [9.88, 24.58]	0.721
Dbil (μmol/L, M [P25, P75])	6.40 [4.10, 10.10]	6.30 [4.10, 9.70]	7.70 [4.75, 12.25]	<0.001
TP (g/L, M [P25, P75])	55.60 [48.50, 62.90]	55.80 [48.80, 63.00]	54.25 [46.40, 62.23]	0.016
ALB (g/L, M [P25, P75])	31.67 [26.20, 37.00]	31.80 [26.30, 37.10]	30.55 [25.10, 36.55]	0.015
GGT (U/L, M [P25, P75])	28.19 [16.00, 62.00]	28.00 [15.70, 60.00]	36.00 [17.00, 74.25]	0.042
Scr (μmol/L, M [P25, P75])	78.00 [53.00, 130.00]	74.00 [51.00, 118.00]	139.00 [76.00, 222.00]	<0.001
BUN (mmol/L, M [P25, P75])	7.24 [5.22, 10.51]	6.95 [5.08, 10.12]	10.38 [6.99, 14.99]	<0.001
UA (μmol/L, M [P25, P75])	272.00 [173.00, 411.00]	258.00 [170.00, 390.00]	361.00 [245.00, 508.50]	<0.001
Urinary protein, *n* (%)	167 (8.2)	126 (7.1)	41 (15.5)	<0.001
Hb (g/dl, M [P25, P75])	117.00 [98.00, 138.00]	117.00 [98.00, 138.00]	116.50 [96.75, 139.25]	0.844
PLT (K/UL, M [P25, P75])	150.00 [98.00, 214.00]	151.00 [101.00, 217.00]	139.00 [85.75, 200.00]	0.001
WBC (K/UL, M [P25, P75])	12.30 [9.60, 16.18]	12.30 [9.52, 16.00]	12.53 [9.91, 18.25]	0.075
Potassium (mmol/L, M [P25, P75])	3.96 [3.48, 4.47]	3.96 [3.48, 4.47]	3.96 [3.47, 4.50]	0.975
Sodium (mmol/L, M [P25, P75])	143.00 [138.00, 151.00]	143.00 [138.00, 151.00]	143.00 [138.98, 151.12]	0.588
Calcium (mmol/L, M [P25, P75])	2.12 [1.96, 2.24]	2.12 [1.96, 2.25]	2.12 [1.98, 2.22]	0.350
Donation after circulatory death, *n* (%)	1,567 (76.8)	1,349 (75.9)	218 (82.6)	0.021
Recipient variables				
Male recipient, *n* (%)	1,501 (73.5)	1,286 (72.4)	215 (81.4)	0.002
Recipient age (years, M [P25, P75])	36 [30, 45]	37 [30, 45]	36 [30, 44]	0.312
HLA mismatch (numbers, M [P25, P75])	2 [1, 2]	2 [1, 3]	2 [1, 2]	0.434
Recipient BMI (kg/m^2^, M [P25, P75])	21.34 [19.26, 23.66]	21.34 [19.15, 23.53]	21.87 [19.78, 24.04]	0.003
Positive PRA, *n* (%)	143 (7.0)	130 (7.3)	13 (4.9)	0.197
HMP parameters				
Initial flux (mL/min, M [P25, P75])	95.00 [79.00, 108.00]	97.00 [80.00, 110.00]	85.00 [66.00, 100.00]	<0.001
Initial pressure (mmHg, M [P25, P75])	35.00 [30.00, 40.00]	35.00 [30.00, 39.00]	35.00 [35.00, 40.00]	<0.001
Initial resistance (mmHg·mL^−2^·min^−1^, M [P25, P75])	0.35 [0.29, 0.42]	0.34 [0.29, 0.40]	0.40 [0.32, 0.50]	<0.001
Terminal flux (mL/min, M [P25, P75])	110.00 [98.00, 120.00]	110.00 [100.00, 121.00]	101.00 [86.75, 115.25]	<0.001
Terminal pressure (mmHg, M [P25, P75])	30.00 [30.00, 35.00]	30.00 [30.00, 35.00]	35.00 [30.00, 39.00]	<0.001
Terminal resistance (mmHg·mL^−2^·min^−1^, M [P25, P75])	0.24 [0.20, 0.30]	0.24 [0.20, 0.30]	0.28 [0.22, 0.35]	<0.001
Perfusion time (h, M [P25, P75])	8.00 [5.00, 10.00]	8.00 [5.00, 10.00]	8.00 [5.88, 10.00]	0.015

DGF: delayed graft function; BMI: body mass index; CIT: cold ischemia time; WIT: warm ischemia time; ALT: alanine aminotransferase; AST: aspartate aminotransferase; Tbil: total bilirubin; Dbil: direct bilirubin; TP: total protein; ALB: albumin; GGT: gamma-glutamyl transferase; Scr: serum creatinine; BUN: blood urea nitrogen; UA: uric acid; Hb: hemoglobin; PLT: platelets; WBC: white blood cells; HLA: human leukocyte antigen; PRA: panel reactive antibodies; HMP: hypothermic machine perfusion.

According to univariate LR analysis (Table S2), a longer CIT and WIT of the donor kidney, higher levels of ALT, AST, Dbil, Scr, BUN, and UA in donors, as well as a higher BMI in recipients, were identified as risk factors for DGF. Conversely, higher levels of TP, ALB, and PLT in donors were protective factors. Additionally, a history of hypertension in donors, positive urinary protein, donors from DCD, and male recipient sex were associated with an increased risk of DGF. Regarding the primary disease of donors, cerebrovascular disease was identified as a risk factor for DGF compared to craniocerebral trauma and tumor.

### 3.2. Association between HMP parameters and graft function

Significant differences in HMP parameters were identified between the DGF and non-DGF groups (Table 1). The non-DGF group exhibited higher initial and terminal flux, while the DGF group demonstrated higher pressure, resistance, and perfusion time (*p* < 0.05). Univariate LR analysis identified higher pressure, resistance, and perfusion time as risk factors for DGF, whereas a higher flux was identified as a protective factor (Table S2).

**Figure 2.**
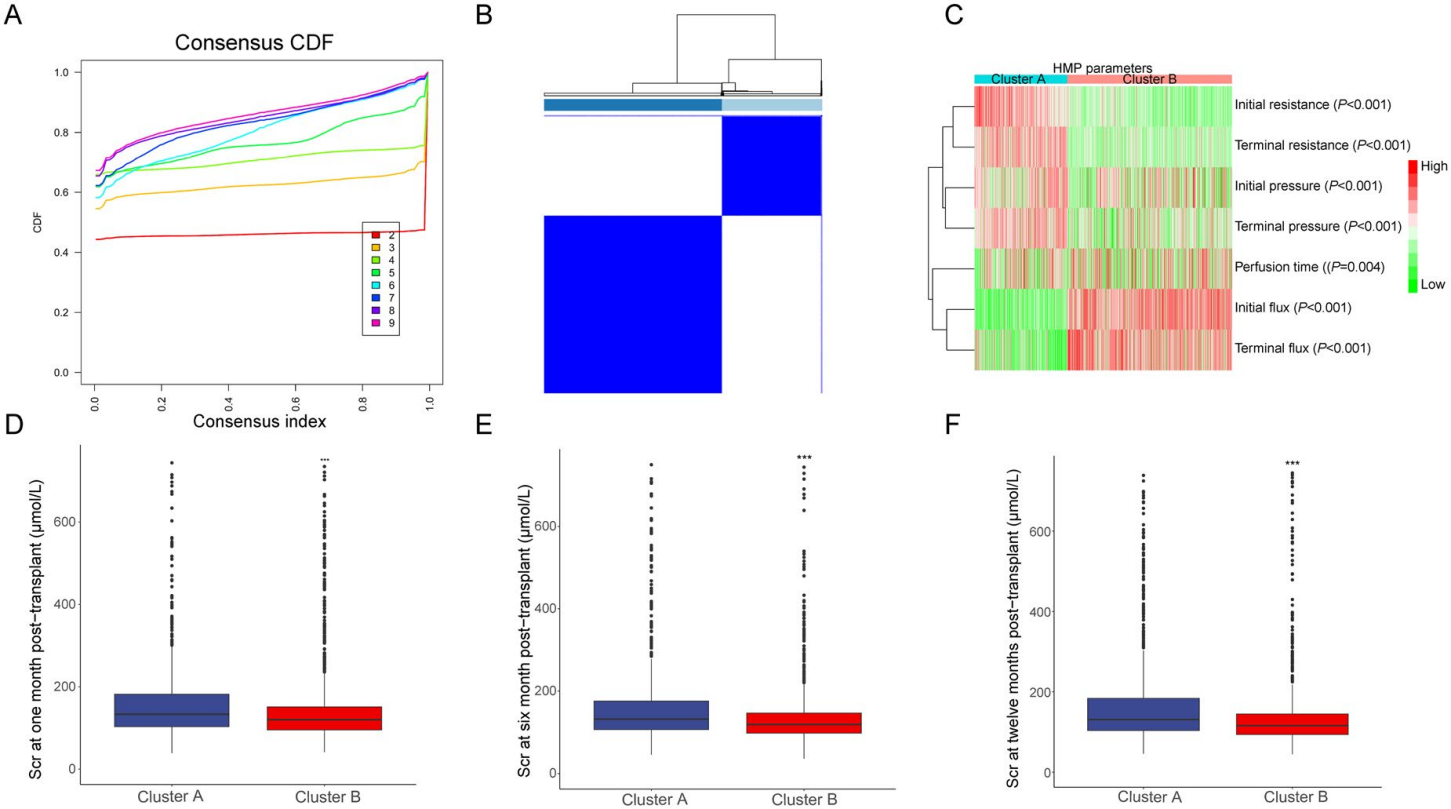
Consensus clustering of recipients according to HMP parameters. (A) CDF curves generated from consensus clustering at different cluster numbers (*k* = 2–9). The algorithm assesses cluster stability by repeatedly subsampling the dataset and reassigning samples. The CDF curve at *k* = 2 showed the largest increase in clustering stability, whereas higher k values yielded minimal improvement, indicating that two clusters represent the optimal solution. (B) Consensus matrix heatmap for *k* = 2 clusters. Each point represents the consensus value between a pair of samples across multiple resampling, with blue indicating high consensus and white indicating low consensus. Clear block structures along the diagonal indicate stable clustering into two distinct groups. (C) The distribution of HMP parameters in Cluster A and Cluster B. The comparison of Scr in recipients at (D) 1, (E) 6, and (F) 12 months after kidney transplantation in Cluster A and Cluster B. CDF: cumulative distribution function; Scr: serum creatinine.

To further explore the potential relationships among HMP parameters, graft function, and donor characteristics, a cluster analysis was performed. This analysis identified two optimal clusters based on HMP parameters: Cluster A and Cluster B (Figures 2A,B, Table S3). Cluster A exhibited significantly higher resistance and pressure, whereas Cluster B demonstrated notably shorter perfusion time and higher flux (Figure 2C, *p* < 0.05). Importantly, the incidence of DGF was significantly higher in recipients from Cluster A compared to those in Cluster B (Table S3, 19.5 *vs.* 9.2%, *p* < 0.05). In terms of donor characteristics, Cluster A exhibited higher levels of Scr, BUN, and UA (*p* < 0.05). Additionally, donors in Cluster A had a greater prevalence of hypertension history, a higher proportion of DCD, and a longer time of WIT (*p* < 0.05). Follow-up rates for postoperative Scr levels were 99.1, 94.4, and 90.4% for 1, 6, and 12 months, respectively. Scr levels in Cluster A were significantly higher than those in Cluster B at each postoperative time point (Figures 2D–F, Table S3, *p* < 0.05).

**Figure 3.**
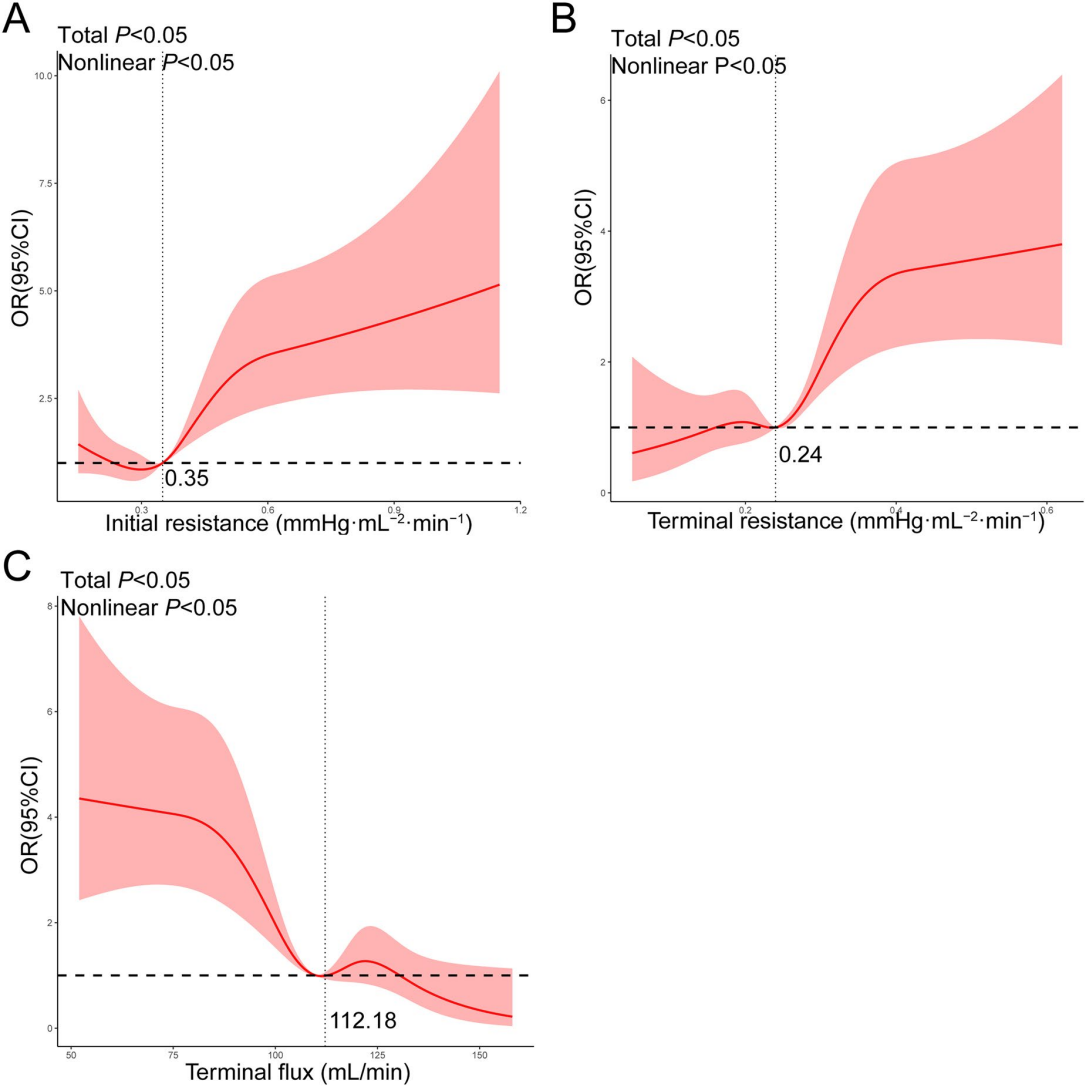
The non-linear relationship of DGF with (A) initial resistance, (B) terminal resistance, and (C) terminal flux visualized by RCS. RCS: restricted cubic spline.

Using RCS analysis, a non-linear relationship between HMP parameters and DGF risk was identified (Figure 3, *p* < 0.05). Threshold effects were observed, with inflection points at 0.35 and 0.24 mmHg·mL^−2^·min^−1^ for initial and terminal resistance, respectively. Below these thresholds, DGF risk remained constant or decreased, whereas values above the thresholds were associated with a rapid increase in risk. DGF risk increased when terminal flux was below 112.18 mL/min but decreased rapidly above this threshold. Based on these thresholds, recipients were divided into two groups, and Scr levels were compared at one, six, and twelve months postoperatively (Figure S1). Scr levels at all postoperative time points were significantly elevated when initial or terminal resistance exceeded their respective thresholds (*p* < 0.05). Conversely, recipients with terminal flux values below the threshold also demonstrated significantly higher Scr levels at the same time points.

### 3.3. Feature selection in the train set

**Figure 4.**
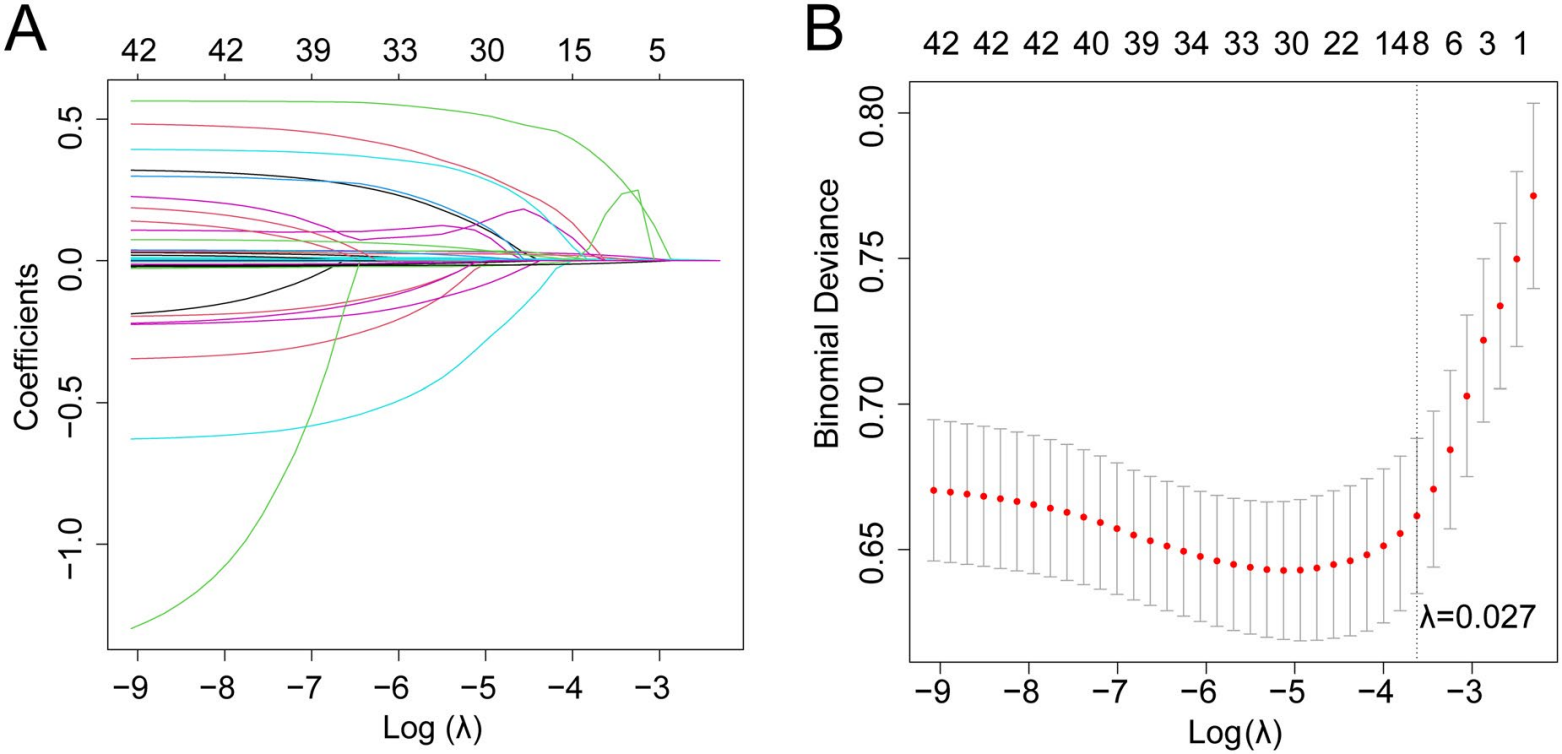
Feature selection based on the LASSO-penalized LR algorithm in the train set. (A) Coefficient profiles of the candidate predictors plotted against log (*λ*), where *λ* is the tuning parameter controlling the degree of shrinkage. As *λ* increases, more coefficients shrink toward 0, leaving only the most important predictors. (B) Ten-fold cross-validation for selecting the optimal *λ* value, with the vertical axis representing the mean squared error of prediction. The vertical dashed line indicates the *λ* value chosen according to the 1-standard error rule, which results in a more parsimonious model while maintaining good predictive performance. LR: logistic regression.

The dataset was split into train and test sets in a 7:3 ratio to facilitate model development and evaluation. Clinical characteristics and DGF incidence were comparable between the train and test sets, with no significant differences that could bias the selection process (Table S4, *p* > 0.05). Subsequent feature selection was conducted on the train set. To identify key predictors of DGF, we applied the Lasso-penalized LR algorithm. This approach selects the most relevant variables while minimizing overfitting by shrinking less important variable coefficients to 0, thereby excluding them from the model. Figure 4A illustrates the variability of coefficients among selected variables, demonstrating the selection dynamics as *λ* changes. To optimize model performance, 10-fold cross-validation was employed on the train set to fine-tune the *λ* parameter, balancing predictive accuracy with model simplicity. An optimal *λ* value of 0.027 was determined, yielding a model with high predictive power and a minimal set of variables. At this threshold, seven variables were selected, including history of hypertension, ALT, AST, Scr, BUN, terminal flux, and terminal pressure, as shown in Figure 4B.

After constructing the multivariable LR model based on these selected variables, we found that although ALT and AST were significant factors in the univariate LR analysis (Table S2, *p* < 0.05), they were not significant in the multivariable model (Table S5, *p* > 0.05). VIF analysis revealed that both ALT (VIF: 2.92) and AST (VIF: 3.01) had higher multicollinearity than other variables, which was further supported by correlation analysis showing a strong association between them (Figure S2, *r* = 0.67, *p* < 0.05). To refine the model, we individually assessed the predictive performance of ALT and AST for predicting DGF, finding that AST (0.58, 95% CI: 0.53–0.63) had a higher AUC than ALT (0.57, 95% CI: 0.52–0.62). Consequently, ALT was excluded from the final model, and only AST was retained. With this adjustment, all included variables in the final model achieved statistical significance (Table 2, *p* < 0.05), resulting in a more robust and interpretable predictive model.

**Table 2.** Variables selected for predicting DGF.

Variables	*β*	OR	*p*-Value	VIF
Intercept	−7.35	0.00	<0.001	/
History of hypertension (Yes/No)	0.89	2.44	<0.001	1.09
AST (U/L)	0.20	1.23	0.002	1.20
Scr (μmol/L)	0.47	1.61	<0.001	1.45
BUN (mmol/L)	0.52	1.68	<0.001	1.37
Terminal flux (mL/min)	−0.02	0.98	<0.001	1.19
Terminal pressure (mmHg)	0.03	1.03	0.041	1.23

OR: odds ratio; VIF: variance inflation factor.

### 3.4. Evaluation of model performance

**Figure 5.**
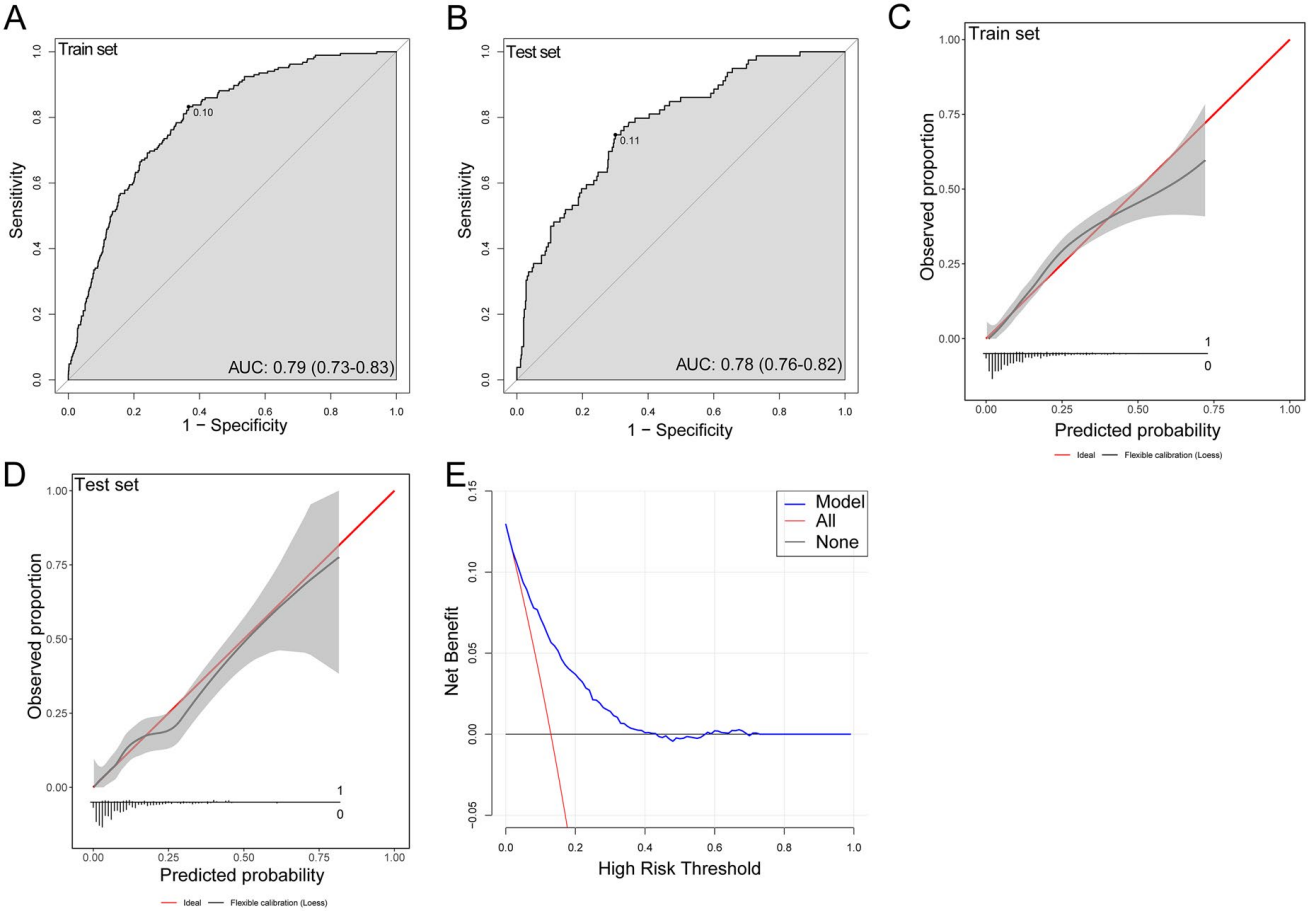
The predictive model’s performance was evaluated *via* discrimination, calibration, and clinical utility. ROC curve analysis of the LR model in the (A) train and (B) test sets. Calibration curves of the LR model in the (C) train and (D) test sets. (E) Clinical decision curve analysis of the predictive model. ROC: receiver operating characteristic; AUC: the area under the curve.

Based on the selected features, an LR model was developed to predict the risk of DGF in transplant recipients (Table 2). Risk scores were calculated using the regression coefficients of each variable, including the model’s intercept. Model performance was assessed in both the train and test sets. The model achieved an AUC of 0.79 (95% CI: 0.73–0.83) in the train set and 0.78 (95% CI: 0.76–0.82) in the test set, indicating good discriminatory performance (Figures 5A,B). Overall model fit was further quantified using Nagelkerke’s pseudo-*R*^2^, which was 0.22, reflecting a meaningful improvement over the null model.

At the optimal cutoff determined by the Youden index, the sensitivity and specificity in the validation set were 0.75 and 0.70, respectively, indicating a balanced classification performance. To assess the robustness of variable selection, sensitivity analyses were performed using reduced models excluding either Scr or BUN (Figure S3). The model excluding Scr showed a discriminative performance comparable to the primary model but with reduced sensitivity, whereas the model excluding BUN demonstrated a lower AUC, indicating inferior overall discrimination. These findings support the inclusion of both variables in the final model.

Calibration curves were constructed to further assess model reliability. The results demonstrated strong agreement between predicted probabilities and actual outcomes, supporting the robustness of the model’s predictions (Figures 5C,D). Moreover, DCA was performed to evaluate the potential clinical benefit of the model. The results demonstrated a high net benefit across a wide range of threshold probabilities (Figure 5E), suggesting potential utility for risk stratification.

### 3.5. Nomogram and web-based tool for visualization

**Figure 6.**
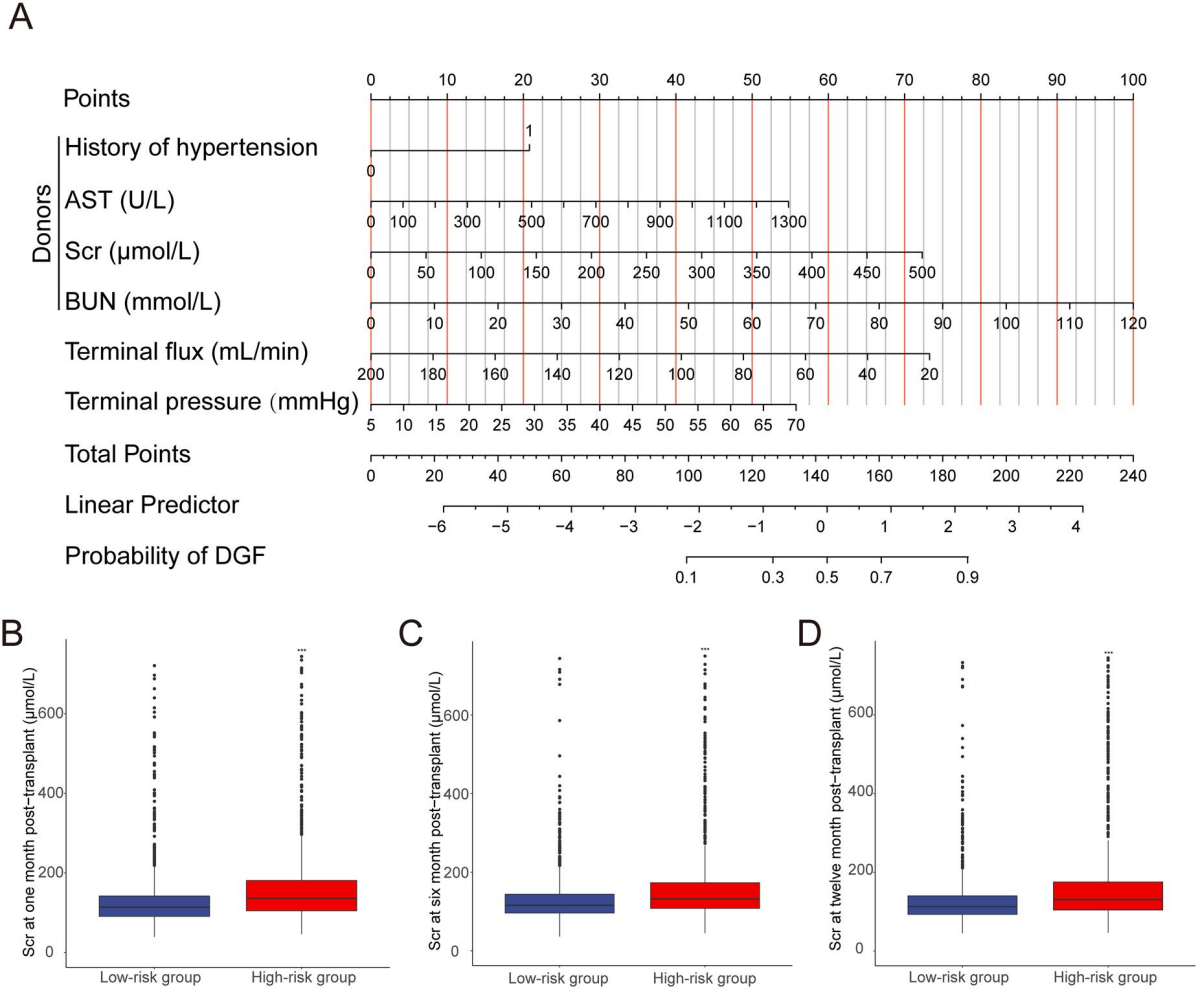
Nomogram for predicting DGF and associated Scr levels. (A) Nomogram of the LR model for predicting DGF. Each predictor variable (history of hypertension, AST, Scr, BUN, terminal flux, and terminal pressure) is assigned a point value according to its contribution to the risk of DGF. Total points are calculated by summing the individual points, which are then mapped to the linear predictor and the corresponding probability of DGF. (B–D) Comparison of Scr levels between low-risk and high-risk groups at (B) 1, (C) 6, and (D) 12 months post-transplantation. AST: aspartate aminotransferase; BUN: blood urea nitrogen.

To improve clinical applicability, the LR model was visualized as a nomogram (Figure 6A). For each recipient, the model-derived variable scores were summed to obtain a total score. A vertical line was then extended from the total score to the probability axis, determining the predicted probability of DGF. The nomogram provided a more intuitive alternative to mathematical equations for clinical use. Risk scores for DGF were calculated using the nomogram, and recipients were stratified into high-risk and low-risk groups based on the median risk score. Recipients in the high-risk group exhibited consistently higher Scr levels than those in the low-risk group throughout the first postoperative year (Figures 6B,D, *p* < 0.05). These findings suggest that the predictive model may also provide supportive information for early postoperative graft function surveillance.

In addition to the nomogram, we further developed a web-based calculator that allows clinicians to directly input the relevant variables and obtain the individualized probability of DGF (https://boqingdong.shinyapps.io/XJTU_KT_DXM__DGF/). To facilitate broader accessibility and practical use at the bedside, we also generated a QR code linked to the online calculator, enabling users to conveniently access the prediction tool *via* mobile devices (Figure S4). This digital extension of the model aims to facilitate visualization and exploratory clinical use.

## 4. Discussion

Over the past decade, the growing disparity between the rising demand for KT among ESRD patients and the constrained supply of donor organs has led to an increased reliance on expanded criteria donors [19,20]. As a result, optimizing organ preservation strategies and establishing robust quality assessment systems have become essential. Compared to SCS, HMP has been shown to significantly improve graft viability while concurrently offering valuable physiological parameters for transplant assessment. However, previous studies exploring the relationship between HMP parameters and graft function have often been limited by small sample sizes and have frequently neglected potential nonlinear associations [21]. In this largest single-center study to date, we examined the association between HMP parameters and graft function in DDKT recipients. We demonstrated that even modest changes in resistance, pressure, or flux—while overlapping between DGF and non-DGF kidneys—were statistically robust and potentially clinically meaningful, reflecting subtle microvascular injury or compromised organ quality. By applying RCS analysis, we revealed nonlinear relationships between HMP parameters and DGF risk. Furthermore, unsupervised clustering identified donor subgroups with distinct DGF incidences and long-term graft function trajectories, linking early perfusion characteristics with both short- and long-term outcomes. These findings provide new insights into the predictive value of HMP parameters, supporting their use for refined perioperative and early post-transplant risk stratification and clinical management.

DGF is a significant complication in KT, often leading to prolonged hospitalization, increased risk of AR, and reduced long-term graft survival. Importantly, DGF primarily reflects early graft recovery and perioperative injury rather than serving as a determinant of organ acceptance or allocation decisions. According to group comparison and univariate LR analysis, our study identified multiple donor and recipient characteristics associated with DGF risk, many of which have been reported in previous studies. These factors include BUN and Scr levels in donors and higher BMI in both donors and recipients [22,23]. Given the critical role of organ preservation in influencing early graft function, we further evaluated HMP, the most commonly used extracorporeal technique, which continuously circulates a cold preservation solution through the organ. Its parameters—flux, resistance, pressure, and perfusion time—serve as indicators of organ viability and quality. We found that an increase in initial and terminal flux was associated with a reduced risk of DGF, whereas higher resistance, elevated pressure, and prolonged perfusion time were correlated with an increased incidence of DGF. Physiologically, higher flux likely reflects more efficient microvascular perfusion within the donor kidney, suggesting that the renal microcirculation is largely intact and capable of delivering sufficient oxygen and nutrients to parenchymal cells during preservation. This optimal perfusion may mitigate IRI, limit endothelial cell dysfunction, and reduce the accumulation of metabolic waste products, all of which are critical determinants of early graft function. In contrast, increased resistance and elevated perfusion pressure may indicate compromised microvascular integrity, such as subtle endothelial injury, capillary collapse, or interstitial edema, which can impair oxygen and nutrient delivery and exacerbate ischemic injury. Prolonged perfusion time under these adverse hemodynamic conditions may further stress the organ, contributing to the development of DGF. Weberskirch et al. analyzed 45 recipients of HMP kidneys and found that kidneys from donors whose recipients developed DGF had lower flux values [12]. In a larger cohort of 581 recipients, Singh and colleagues reported that higher flux was significantly associated with lower Scr on postoperative day 5 [24]. Similarly, Parikh et al. conducted a prospective study and observed that flux at 1 h after HMP was negatively correlated with DGF, while higher flux predicted better six-month graft function, as reflected by higher eGFR [25]. Moreover, Kolonko and coworkers prospectively analyzed 364 kidney transplant recipients and demonstrated that higher early post-transplant resistance index values were linked to worse graft function, along with increased risks of graft loss and patient death during long-term follow-up [26]. Furthermore, Patel et al. retrospectively studied 73 pump-perfused deceased donor kidneys and showed that elevated perfusion pressure was associated with increased vascular resistance and a higher incidence of DGF [27]. Taken together, both our results and prior evidence highlight that HMP-derived hemodynamic parameters are not merely passive reflections of organ status but active physiological markers that capture the integrity of donor kidney microcirculation. Careful monitoring and interpretation of these parameters may therefore provide valuable guidance for optimizing preservation strategies and improving post-transplant outcomes.

Unsupervised machine learning techniques are widely employed in cancer research, especially for high-throughput data analysis. However, the application of unsupervised machine learning in DDKT-related clinical research remains limited, and no studies have utilized unsupervised machine learning with HMP parameters. Given the resemblance between HMP parameters and high-throughput data, applying unsupervised learning to HMP parameters for donor characterization and DGF risk stratification in recipients is a logical approach. In this study, recipients were divided into two clusters based on HMP parameters. Recipients in Cluster A exhibited higher initial and terminal resistance and pressure, along with adverse donor factors such as elevated Scr, BUN, and UA levels. Additionally, in recipients of Cluster A, those with higher proportion of history of hypertension indicated that HMP parameters may serve as indicators of donor kidney microcirculation, as hypertension is known to significantly impair microvascular function. Importantly, Cluster A recipients had a significantly higher risk of DGF than those in Cluster B, underscoring the potential prognostic value of HMP parameters in early predicting post-transplant outcomes. Subsequently, this study elucidated the non-linear relationship between HMP parameters and DGF risk, unveiling potential threshold effects. Specifically, when both initial and terminal resistances exceeded the threshold or when terminal flux dropped below the threshold, the risk of DGF increased substantially. Most existing studies have primarily focused on the association between HMP parameters and DGF, with only a few small-sample studies evaluating their relationship with post-KT graft function [21,28]. To our knowledge, this study represents one of the most comprehensive investigations of the relationship between HMP parameters and postoperative graft function. In Cluster A, characterized by high resistance, high pressure, and low flux, recipients had significantly higher Scr levels at one, six, and twelve months postoperatively compared to those in Cluster B. Moreover, when initial and terminal resistance exceeded the threshold or terminal flux fell below the threshold, recipients demonstrated significantly elevated Scr levels within the first year after transplantation. These findings suggest that HMP parameters may provide early signals associated with subsequent graft function trajectories, linking early graft status with longer-term outcomes. By integrating initial and terminal HMP parameters, risk stratification can be utilized to assess the likelihood of post-transplant DGF and long-term graft function. This assessment can be performed at multiple stages, including organ procurement, preservation, and pre-transplant evaluation. When the donor kidney HMP parameters indicate high risk and additional risk factors such as a history of hypertension and DCD origin are present, recipient selection and immunosuppressive strategy may warrant careful consideration within the context of maximizing graft utilization rather than excluding marginal organs. However, decisions regarding kidney discard should be made cautiously, considering the ongoing critical shortage of donor organs [29]. Further pathological evaluation is necessary to guide these decisions [30,31]. For recipients of high-risk kidneys, close monitoring for DGF is warranted, and frequent postoperative follow-up is recommended to mitigate long-term graft dysfunction. Subsequent research should integrate HMP parameters with biomarkers and early graft function recovery in recipients. Combining these factors with long-term graft survival will contribute to establishing a unified definition of DGF.

Currently, there are no specific or early treatments for DGF. If DGF is diagnosed immediately after KT, clinicians can initiate personalized care earlier, including adjustments to the initiation and dosage of CNI [32]. Therefore, this study developed a predictive model using a large single-center dataset to estimate the likelihood of DGF before transplantation. This model is intended as an internally validated, center-specific risk stratification tool to support early clinical decision-making. Six variables were identified as statistically significant in both univariate and multivariate logistic regression analyses. This indicates that these variables are independently associated with DGF, rather than being confounded by other factors. The consistency of significance across analyses underscores their strong predictive value and independent contribution to DGF risk. Furthermore, the stability of their effect sizes suggests minimal multicollinearity, reinforcing the model’s robustness and interpretability. This model achieved strong predictive performance, with an AUC of 0.79 in the train set and 0.78 in the test set. Calibration curves and concordance indices further support the model’s reliability and discriminative power, demonstrating consistent predictive accuracy across datasets. To enhance its clinical utility, we visualized the multivariable LR model as a nomogram, providing a user-friendly tool for estimating individual DGF risk scores. In addition, we developed a web-based calculator and generated a QR code to facilitate convenient use on mobile devices, further improving accessibility in real-world clinical settings. Notably, the observed association between high-risk scores and elevated post-transplant Scr levels served as a practical indicator for graft function monitoring, underscoring the model’s potential role as a decision-support tool in postoperative management.

Following organ procurement, kidneys undergo a cascade of physiological and pathological alterations during machine perfusion (MP). Despite the protective effect of continuous machine circulation, residual ischemic stress persists, leading to mitochondrial dysfunction, impaired ATP generation, and disruption of ionic homeostasis within tubular epithelial cells [33]. Endothelial activation and glycocalyx shedding contribute to microvascular instability, while oxidative stress and the accumulation of reactive oxygen species further aggravate tubular apoptosis and necrosis [34]. These cellular and subcellular injuries result in the leakage of molecules such as lactate dehydrogenase (LDH), glutathione S-transferase (GST), and neutrophil gelatinase-associated lipocalin (NGAL) into the perfusate, which have been explored as surrogate biomarkers of graft injury and viability in perfusion solution (Table S6). Several studies have investigated the diagnostic and prognostic value of these perfusate biomarkers during MP. For instance, Guy et al. conducted a metabolomic analysis of perfusate samples collected at multiple time points during HMP from 26 cadaveric donor kidneys and found that the levels of creatinine, leucine, and gluconate were closely associated with DGF [35]. Similarly, Hoogland et al. analyzed multiple perfusate biomarkers in 335 DCD kidneys and found that LDH and IL-18 levels were associated with DGF, whereas NGAL showed no significant association [36]. Moreover, Duarte et al. measured soluble DNA in the perfusate and demonstrated that it could predict DGF and was closely correlated with long-term graft function [37]. Furthermore, Hall et al. evaluated GST isoenzymes and showed that pi-GST was an independent risk factor for DGF, whereas α-GST was not. However, previous biomarker studies exhibit several limitations [38]. Most were single-center studies with small sample sizes, and their findings were often inconsistent across centers [36]. For example, in the study by Moers et al. GST, N-acetyl-β-D-glycosaminidase, and H- fatty acid-binding protein (H-FABP) levels in the perfusate during HMP were identified as independent predictors of DGF (*n* = 306), whereas in another larger cohort (*n* = 335), GST and H-FABP showed no significant association with DGF [36,39]. Moreover, many of these studies were primarily descriptive and did not establish predictive models, but merely evaluated the association between individual biomarkers and DGF, without incorporating perfusion-derived parameters or adjusting for relevant clinical variables [40,41]. Even in the few studies that did construct predictive models, they were often limited by small cohorts and lacked appropriate division into training and validation datasets, thereby reducing robustness and increasing the risk of overfitting [36,42]. Importantly, many of these studies selected biomarkers based on individual molecular events, such as oxidative stress or tubular injury, whereas organ preservation involves multiple concurrent processes, including programmed cell death, energy metabolism alterations, and other subcellular responses [43]. Moreover, the use of biomarker assays increases cost and complexity, introduces batch effects, and lacks standardized thresholds, which together restrict clinical implementation and external reproducibility [44]. In contrast, the present study provides a complementary and pragmatically oriented approach by leveraging real-time HMP parameters in combination with machine-learning algorithms to assess graft quality. Importantly, our study incorporated a relatively large sample size and explicitly divided the data into training and test cohorts, ensuring robust model evaluation and reducing the risk of overfitting. We applied a multivariate LR model, which is the most widely used parametric model in clinical research due to its interpretability and simplicity, and has also been commonly adopted in previous HMP-related studies [45]. Rather than relying on additional biochemical assays, this approach captures continuous hemodynamic and metabolic dynamics recorded during perfusion, offering a direct and functional evaluation of organ performance within the context of early post-transplant risk stratification. It reduces dependence on the cost, variability, and logistical delay associated with biomarker assays, while maintaining clinically meaningful predictive capability. Furthermore, because these perfusion parameters are automatically and routinely recorded, our approach enhances reproducibility, objectivity, and potential scalability across transplant centers. Importantly, HMP parameters reflect the overall functional state of the organ resulting from multiple concurrent molecular and cellular events during perfusion, whereas traditional perfusate biomarkers tend to emphasize specific molecular processes, such as oxidative stress or tubular injury. Looking forward, future multicenter studies integrating both molecular and functional dimensions—combining perfusate biomarkers that indicate cellular injury with perfusion-derived parameters reflecting physiological performance—may offer a more comprehensive and biologically grounded assessment of graft viability. In addition, future research should further explore whether the profiles and predictive values of perfusate biomarkers differ under distinct perfusion temperatures and oxygenation conditions. Incorporating such multimodal data, together with donor and clinical variables, into unified predictive frameworks could significantly improve model robustness, interpretability, and translational utility in optimizing graft assessment and predicting post-transplant outcomes [46].

While our study demonstrated a non-linear relationship between HMP parameters and DGF risk and developed an LR model with good predictive performance, several limitations should be noted. First, this study did not include biomarkers of graft injury in the perfusate or histopathological scores, which could have provided complementary molecular and structural insights and enhanced understanding of graft injury mechanisms. Future multicenter studies are warranted to systematically integrate perfusion parameters with molecular and other multimodal biomarkers, enabling the development of standardized, robust predictive models for DGF. Second, although CIT was analyzed, more detailed perioperative intervals, including the time from *in situ* cold perfusion to nephrectomy and vascular anastomosis duration, were unavailable, therefore limiting the evaluation of ischemic injury. Regarding vascular anatomy, polar arteries were routinely reconstructed and connected *via* a standardized Sealring cannula, ensuring complete perfusion; consequently, this uniform approach may explain the lack of association with DGF but limits assessment of anatomical variability elsewhere. Moreover, all grafts were preserved with UW solution followed by non-oxygenated hypothermic perfusion with KPS-1, reducing intra-cohort variability but precluding comparisons with other preservation strategies. Finally, the retrospective and single-center nature of this study inevitably introduces certain limitations. In addition, the relatively low incidence of DGF observed in our cohort, despite a high proportion of DCD donors, likely reflects center-specific factors such as relatively short ischemia times, moderate recipient age, and the routine use of hypothermic machine perfusion. These favorable conditions may influence baseline risk distributions and model performance, and thus caution is warranted when extrapolating the results to settings with longer ischemia times, different preservation protocols, or higher baseline DGF rates. On the other hand, the single-center setting restricts the diversity of both patient and donor populations, as well as clinical management strategies, which may reduce the external validity of our findings. In addition, the absence of external validation, especially from multi-center perspective cohorts, limits the ability to fully assess the reproducibility and clinical applicability of our model. Therefore, further studies incorporating heterogeneous populations and standardized protocols across multiple institutions are essential to confirm the robustness of our conclusions and to support broader clinical implementation.

## 5. Conclusion

In conclusion, this study emphasized the importance of donor and recipient characteristics and HMP parameters in predicting DGF risk. By analyzing a large cohort of KT recipients, we identified critical variables associated with graft function and developed a predictive model with strong discrimination and clinical utility. The nomogram derived from this model offers an accessible tool for clinicians to estimate individualized risk and guide decision-making in recipient management. Further multicenter validation and refinement of predictive models incorporating donor, recipient, and preservation-related factors are required to confirm generalizability across diverse clinical settings and optimize post-transplant outcomes.

## Supplementary Material

Supplemental Material

Supplemental Material

Supplemental Material

Supplemental Material

## Data Availability

All data supporting the findings of this study are available from the corresponding author upon reasonable request.
