# Supplementary figures and images for: Association between hypothermic machine perfusion parameters and graft function in deceased donor kidney transplantation
Source: Ann Med. 2026 Feb 25;58(1):2634488. doi: 10.1080/07853890.2026.2634488 (PMC12943820; doi:10.1080/07853890.2026.2634488)

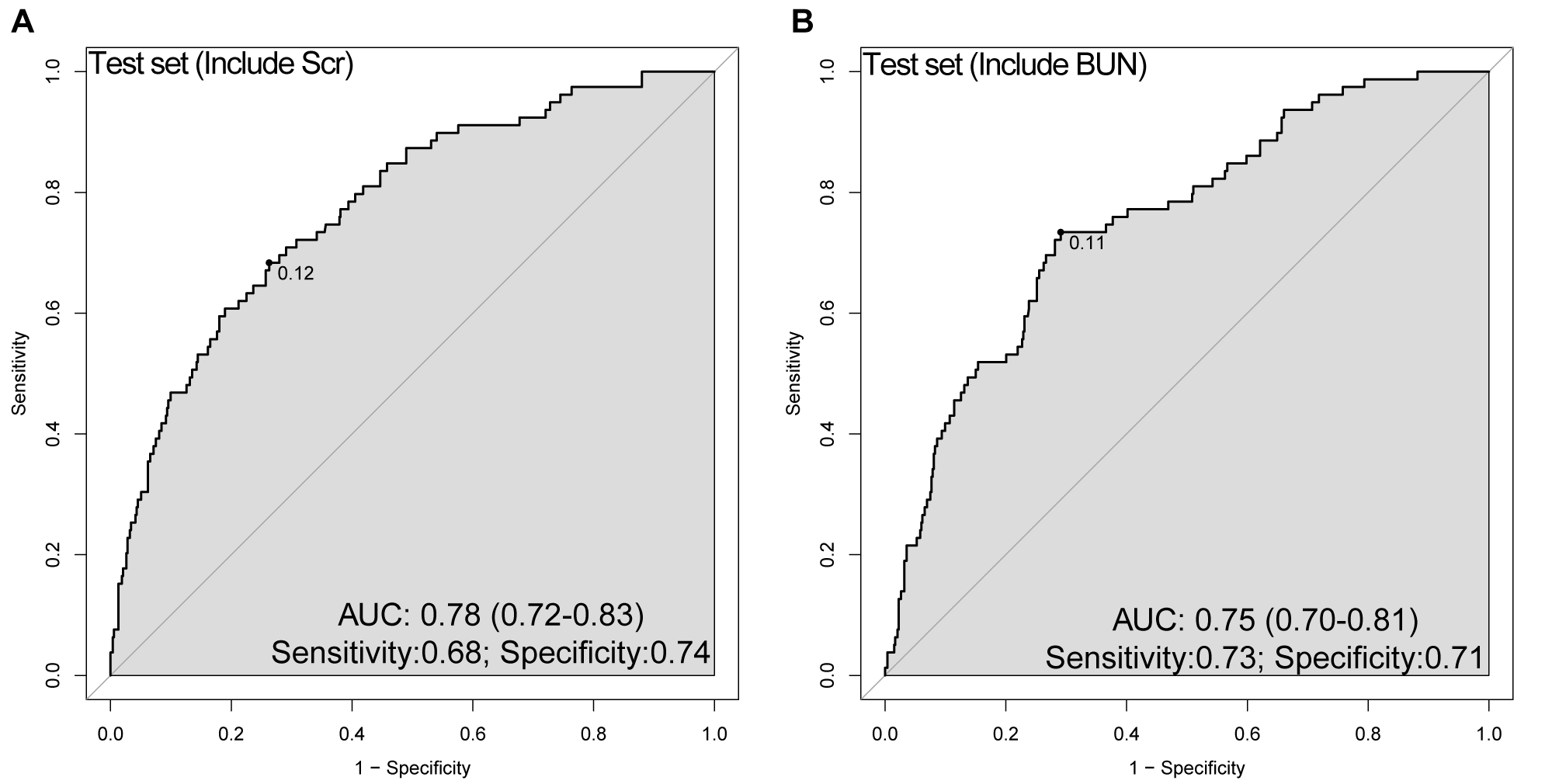

Supplement: Supplemental Material [file IANN_A_2634488_SM7388.tif]

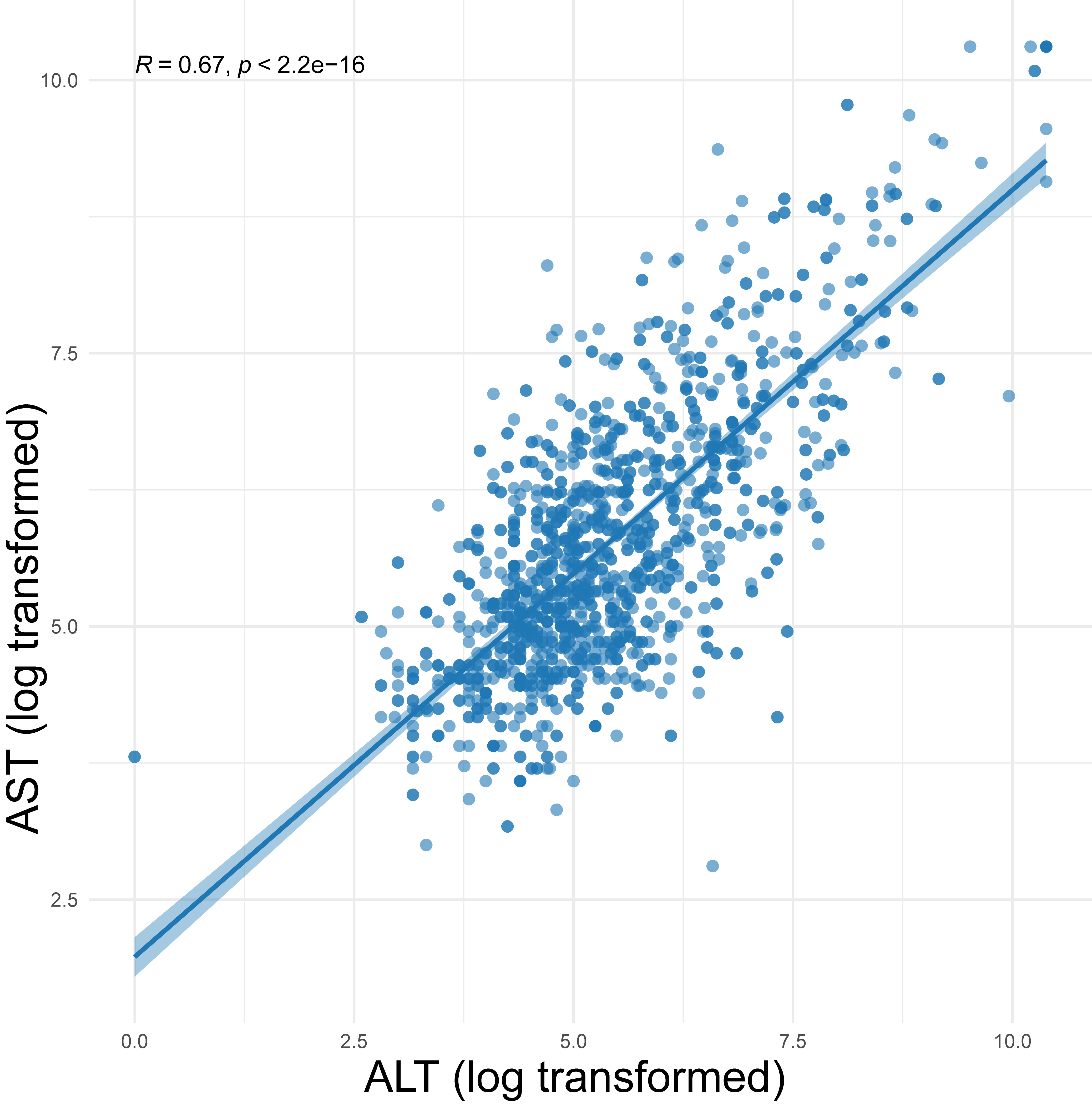

Supplement: Supplemental Material [file IANN_A_2634488_SM7385.tif]

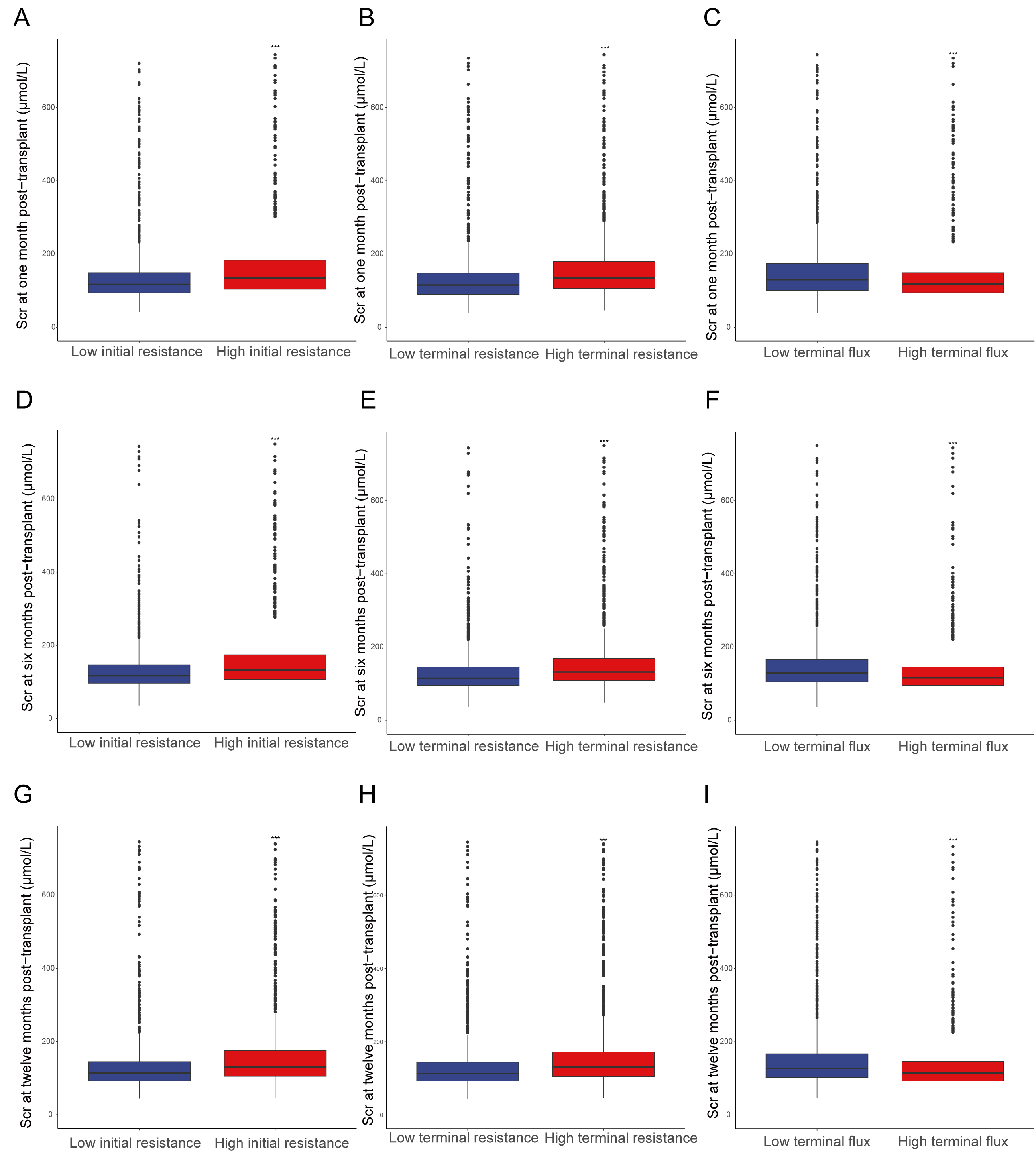

Supplement: Supplemental Material [file IANN_A_2634488_SM7378.tif]
