# Supplementary material for: Association between hypothermic machine perfusion parameters and graft function in deceased donor kidney transplantation
Source: Ann Med. 2026 Feb 25;58(1):2634488. doi: 10.1080/07853890.2026.2634488 (PMC12943820; doi:10.1080/07853890.2026.2634488)
Supplement: Supplemental Material [file IANN_A_2634488_SM7050.docx]

**Table S1.** R packages used in this study.

| **Package** | **Version** | **Method** |
| --- | --- | --- |
| tidyverse | 2.0.0 | Data cleansing and visualization |
| tableone | 0.13.2 | Generate a baseline table |
| ConsensusClusterPlus | 1.62.0 | Perform consensus clustering |
| rms | 6.7.1 | Perform restricted cubic spline and generate nomogram |
| glmnet | 4.1.8 | Perform Lasso algorithm |
| pheatmap | 1.0.12 | Draw pheatmap |
| rmda | 1.6 | Generate decision curve analysis |
| pROC | 1.18.5 | Draw receiver operating characteristic curve |
| CalibrationCurves | 2.0.0 | Draw calibration curve |
| pscl | 1.5.9 | Caculate Nagelkerke R² |

**Table S2.** Univariate logistic regression analysis of DGF.

| **Variable** | **OR** | **5% CI** | **95% CI** | ***P-*value** | ***FDR-adjusted P- value*** |
| --- | --- | --- | --- | --- | --- |
| **Donor variables** |  |  |  |  |  |
| Right donor kidney | 1.23 | 0.95 | 1.60 | 0.115 | 0.164 |
| Polar artery | 1.18 | 0.86 | 1.62 | 0.296 | 0.377 |
| Male donor | 0.98 | 0.71 | 1.36 | 0.890 | 0.890 |
| Donor BMI | 1.04 | 0.99 | 1.08 | 0.092 | 0.136 |
| Donor age | 1.01 | 1.00 | 1.02 | 0.170 | 0.226 |
| Primary disease |  |  |  |  |  |
| Cerebrovascular disease (Reference) | / | / | / | / |  |
| Craniocerebral trauma | 0.55 | 0.40 | 0.73 | *P*<0.001 | *P<*0.001 |
| Hypoxic ischemic encephalopathy | 1.53 | 0.92 | 2.47 | 0.092 | 0.136 |
| Tumor | 0.44 | 0.23 | 0.76 | 0.006 | 0.011 |
| Others | 0.35 | 0.02 | 1.77 | 0.317 | 0.388 |
| History of hypertension | 2.34 | 1.79 | 3.10 | *P*<0.001 | *P<*0.001 |
| CIT | 1.05 | 1.01 | 1.10 | 0.009 | 0.017 |
| WIT | 1.05 | 1.01 | 1.08 | 0.010 | 0.019 |
| ALT | 1.29 | 1.19 | 1.41 | *P*<0.001 | *P<*0.001 |
| AST | 1.33 | 1.21 | 1.46 | *P*<0.001 | *P<*0.001 |
| Tbil | 1.02 | 0.88 | 1.17 | 0.830 | 0.851 |
| Dbil | 1.29 | 1.15 | 1.45 | *P*<0.001 | *P<*0.001 |
| TP | 0.67 | 0.49 | 0.94 | 0.017 | 0.030 |
| ALB | 0.59 | 0.42 | 0.84 | 0.003 | 0.006 |
| GGT | 1.08 | 0.99 | 1.18 | 0.069 | 0.110 |
| Scr | 2.43 | 2.08 | 2.85 | *P*<0.001 | *P*<0.001 |
| BUN | 2.45 | 2.06 | 2.92 | *P*<0.001 | *P*<0.001 |
| UA | 1.93 | 1.63 | 2.30 | *P*<0.001 | *P*<0.001 |
| Urinary protein | 2.41 | 1.63 | 3.49 | *P*<0.001 | *P*<0.001 |
| Hb | 0.82 | 0.60 | 1.15 | 0.244 | 0.314 |
| PLT | 0.81 | 0.73 | 0.92 | *P*<0.001 | 0.001 |
| WBC | 1.08 | 0.92 | 1.27 | 0.357 | 0.408 |
| Potassium | 1.12 | 0.74 | 1.72 | 0.590 | 0.656 |
| Sodium | 1.17 | 0.35 | 3.82 | 0.797 | 0.839 |
| Calcium | 0.88 | 0.51 | 1.56 | 0.657 | 0.711 |
| Donation after circulatory death | 1.50 | 1.08 | 2.13 | 0.017 | 0.030 |
| **Recipient variables** |  |  |  |  |  |
| Male recipient | 1.68 | 1.22 | 2.35 | 0.002 | 0.005 |
| Recipient age | 0.99 | 0.98 | 1.01 | 0.320 | 0.388 |
| HLA mismatch | 0.94 | 0.81 | 1.07 | 0.344 | 0.405 |
| Recipient BMI | 1.06 | 1.02 | 1.11 | 0.003 | 0.006 |
| Positive PRA | 0.66 | 0.35 | 1.13 | 0.158 | 0.218 |
| **HMP parameters** |  |  |  |  |  |
| Initial flux | 0.98 | 0.98 | 0.99 | *P*<0.001 | *P*<0.001 |
| Initial pressure | 1.10 | 1.06 | 1.13 | *P*<0.001 | *P*<0.001 |
| Initial resistance | 12.03 | 5.92 | 24.72 | *P*<0.001 | *P*<0.001 |
| Terminal flux | 0.97 | 0.97 | 0.98 | *P*<0.001 | *P*<0.001 |
| Terminal pressure | 1.09 | 1.07 | 1.12 | *P*<0.001 | *P*<0.001 |
| Terminal resistance | 51.53 | 14.73 | 180.20 | *P*<0.001 | *P*<0.001 |
| Perfusion time | 1.04 | 1.00 | 1.09 | 0.031 | 0.048 |

OR: odds ratio; CI: confidence interval; DGF: delayed graft function; BMI: body mass index; CIT: cold ischemia time; WIT: warm ischemia time; ALT: alanine aminotransferase; AST: aspartate aminotransferase; Tbil: total bilirubin; Dbil: direct Bilirubin; TP: total protein; ALB: albumin; GGT, gamma-glutamyl transferase; Sc: serum creatinine; BUN: blood urea nitrogen; UA: uric acid; Hb: hemoglobin; PLT: platelets; WBC: white blood cells; HLA: human leukocyte antigen; PRA: panel reactive antibodies; HMP: hypothermic machine perfusion.

**Table S3.** Baseline characteristics of donors in clusters.

|  | **Cluster A** | **Cluster B** | ***P-* value** |
| --- | --- | --- | --- |
| n (%) | 737 (36.1) | 1304 (63.9) |  |
| **Donor variables** |  |  |  |
| Right donor kidney, n (%) | 365 (49.5) | 648 (49.7) | 0.979 |
| Polar artery, n (%) | 168 (22.8) | 217 (16.6) | <0.001 |
| Male donor, n (%) | 568 (77.1) | 1062 (81.4) | 0.021 |
| Donor BMI (kg/m^2^, M [P25, P75]) | 22.49 (20.76, 24.49) | 22.86 (21.11, 24.49) | 0.059 |
| Donor age (years, M [P25, P75]) | 53 (44, 61) | 52 (43, 60) | 0.133 |
| Primary disease, n (%) |  |  | 0.138 |
| Cerebrovascular disease | 377 (51.2) | 609 (46.7) | 0.227 |
| Craniocerebral trauma | 270 (36.6) | 494 (37.9) | 0.601 |
| Hypoxic ischemic encephalopathy | 29 (3.9) | 74 (5.7) | 0.227 |
| Tumor | 53 (7.2) | 119 (9.1) | 0.372 |
| Others | 8 (1.1) | 8 (0.6) | 0.227 |
| History of hypertension, n (%) | 407 (55.2) | 613 (47.0) | <0.001 |
| CIT (hours, M [P25, P75]) | 8.0 (6.0, 11.0) | 9.0 (7.0, 11.0) | 0.053 |
| WIT (mins, M [P25, P75]) | 7 (5, 8) | 6 (0, 8) | <0.001 |
| ALT (U/L, M [P25, P75]) | 34.00 (22.00, 79.00) | 39.00 (24.00, 78.25) | 0.103 |
| AST (U/L, M [P25, P75]) | 44.00 (29.00, 91.00) | 50.00 (29.00, 92.00) | 0.118 |
| Tbil (μmol/L, M [P25, P75]) | 15.40 (10.10, 23.40) | 15.70 (11.07, 23.77) | 0.193 |
| Dbil (μmol/L, M [P25, P75]) | 6.40 (4.10, 10.20) | 6.40 (4.20, 9.93) | 0.889 |
| TP (g/L, M [P25, P75]) | 55.60 (48.00, 64.50) | 55.60 (48.80, 62.12) | 0.650 |
| ALB (g/L, M [P25, P75]) | 32.40 (26.00, 38.50) | 31.00 (26.30, 36.42) | 0.055 |
| GGT (U/L, M [P25, P75]) | 28.00 (15.00, 62.00) | 29.00 (16.00, 61.25) | 0.082 |
| Scr (μmol/L, M [P25, P75]) | 87.00 (57.00, 142.00) | 74.00 (51.00, 122.00) | <0.001 |
| BUN (mmol/L, M [P25, P75]) | 7.44 (5.35, 10.95) | 7.09 (5.15, 10.37) | 0.006 |
| UA (μmol/L, M [P25, P75]) | 297.00 (202.00, 421.00) | 257.00 (164.75, 404.50) | <0.001 |
| Urinary protein, n (%) | 67 (9.1) | 100 (7.7) | 0.297 |
| Hb (g/dl, M [P25, P75]) | 118.00 (99.00, 139.00) | 116.00 (96.75, 138.00) | 0.218 |
| PLT (K/UL, M [P25, P75]) | 150.00 (106.00, 211.00) | 150.00 (96.00, 219.00) | 0.490 |
| WBC (K/UL, M [P25, P75]) | 12.30 (9.70, 16.46) | 12.30 (9.45, 15.99) | 0.342 |
| Potassium (mmol/L, M [P25, P75]) | 3.95 (3.48, 4.50) | 3.96 (3.48, 4.46) | 0.874 |
| Sodium (mmol/L, M [P25, P75]) | 142.60 (138.00, 149.90) | 143.00 (137.98, 151.30) | 0.438 |
| Calcium (mmol/L, M [P25, P75]) | 2.12 (1.96, 2.24) | 2.12 (1.96, 2.25) | 0.306 |
| Donation after circulatory death, n (%) | 651 (88.3) | 916 (70.2) | <0.001 |
| **HMP parameters** |  |  |  |
| Initial flux (mL/min, M [P25, P75]) | 72.00 [59.00, 82.00] | 104.00 [96.00, 113.00] | <0.001 |
| Initial pressure (mmHg, M [P25, P75]) | 35.00 [35.00, 40.00] | 35.00 [30.00, 35.00] | <0.001 |
| Initial resistance (mmHg·mL^-2^·min^-1^, M [P25, P75]) | 0.45 [0.38, 0.54] | 0.30 [0.27, 0.35] | <0.001 |
| Terminal flux (mL/min, M [P25, P75]) | 95.00 [82.00, 102.00] | 118.00 [110.00, 125.00] | <0.001 |
| Terminal pressure (mmHg, M [P25, P75]) | 35.00 [30.00, 39.00] | 30.00 [25.00, 35.00] | <0.001 |
| Terminal resistance (mmHg·mL^-2^·min^-1^, M [P25, P75]) | 0.31 [0.26, 0.38] | 0.21 [0.19, 0.25] | <0.001 |
| Perfusion time (hours, M [P25, P75]) | 7.00 [5.00, 10.00] | 8.00 [6.00, 10.00] | 0.004 |
| **Graft function** |  |  |  |
| DGF, n (%) | 144 (19.5) | 120 (9.2) | <0.001 |
| Scr at 1 month postoperatively (μmol/L, M [P25, P75]) | 134.00 [103.00, 184.00] | 120.00 [95.00, 152.50] | <0.001 |
| Scr at 6 months postoperatively (μmol/L, M [P25, P75]) | 133.00 [108.00, 182.00] | 119.00 [99.00, 149.00] | <0.001 |
| Scr at 12 months postoperatively (μmol/L, M [P25, P75]) | 133.00 [104.00, 192.25] | 117.00 [95.00, 147.00] | <0.001 |

**Table S4.** Baseline characteristics in the train and test sets.

|  | **Train set** | **Test set** | ***P-* value** |
| --- | --- | --- | --- |
| n (%) | 1,429 (70%) | 612 (30%) |  |
| DGF, n (%) | 185 (12.9) | 79 (12.9) | 1 |
| **Donor variables** |  |  |  |
| Right donor kidney, n (%) | 713 (49.9) | 300 (49.0) | 0.753 |
| Polar artery, n (%) | 275 (19.2) | 110 (18.0) | 0.542 |
| Male donor, n (%) | 1,125 (78.7) | 505 (82.5) | 0.058 |
| Donor BMI (kg/m^2^, M [P25, P75]) | 22.84 [20.76, 24.49] | 22.49 [21.04, 24.41] | 0.569 |
| Donor age (years, M [P25, P75]) | 52 [43, 60] | 52 [43, 61] | 0.770 |
| Primary disease, n (%) |  |  | 0.968 |
| Cerebrovascular disease | 694 (48.6) | 292 (47.7) | 0.735 |
| Craniocerebral trauma | 532 (37.2) | 232 (37.9) | 1 |
| Hypoxic ischemic encephalopathy | 121 (8.5) | 51 (8.3) | 1 |
| Tumor | 72 (5.0) | 31 (5.1) | 1 |
| Others | 10 (0.7) | 6 (1.0) | 1 |
| History of hypertension, n (%) | 726 (50.8) | 294 (48.0) | 0.273 |
| CIT (hours, M [P25, P75]) | 9.0 [6.0, 11.0] | 9.0 [6.0, 11.0] | 0.382 |
| WIT (mins, M [P25, P75]) | 6 [3, 8] | 6 [4, 8] | 0.923 |
| ALT (U/L, M [P25, P75]) | 38.00 [24.00, 74.00] | 35.00 [23.00, 72.15] | 0.194 |
| AST (U/L, M [P25, P75]) | 48.00 [30.00, 94.00] | 46.00 [29.00, 88.00] | 0.151 |
| Tbil (μmol/L, M [P25, P75]) | 16.00 [10.90, 24.20] | 14.95 [10.20, 22.92] | 0.223 |
| Dbil (μmol/L, M [P25, P75]) | 6.50 [4.20, 10.20] | 6.10 [4.10, 9.70] | 0.366 |
| TP (g/L, M [P25, P75]) | 55.40 [48.60, 62.70] | 55.70 [48.35, 63.45] | 0.484 |
| ALB (g/L, M [P25, P75]) | 31.67 [26.00, 37.00] | 31.69 [26.70, 37.00] | 0.404 |
| GGT (U/L, M [P25, P75]) | 29.00 [16.00, 62.00] | 27.00 [16.00, 62.00] | 0.632 |
| Scr (μmol/L, M [P25, P75]) | 79.00 [53.00, 132.00] | 77.00 [53.00, 129.00] | 0.952 |
| BUN (mmol/L, M [P25, P75]) | 7.26 [5.30, 10.50] | 7.18 [5.12, 10.64] | 0.448 |
| UA (μmol/L, M [P25, P75]) | 272.00 [173.00, 411.00] | 272.00 [173.00, 416.25] | 0.879 |
| Urinary protein, n (%) | 123 (8.6) | 44 (7.2) | 0.326 |
| Hb (g/dl, M [P25, P75]) | 116.00 [97.00, 138.00] | 118.00 [101.00, 138.25] | 0.224 |
| PLT (K/UL, M [P25, P75]) | 149.00 [98.00, 212.00] | 155.50 [101.00, 218.00] | 0.100 |
| WBC (K/UL, M [P25, P75]) | 12.30 [9.51, 16.05] | 12.30 [9.60, 16.48] | 0.594 |
| Potassium (mmol/L, M [P25, P75]) | 3.96 [3.46, 4.47] | 3.96 [3.50, 4.48] | 0.914 |
| Sodium (mmol/L, M [P25, P75]) | 143.00 [138.00, 151.00] | 142.50 [137.00, 150.10] | 0.061 |
| Calcium (mmol/L, M [P25, P75]) | 2.12 [1.95, 2.24] | 2.12 [1.97, 2.25] | 0.393 |
| Donation after circulatory death, n (%) | 1,089 (76.2) | 478 (78.1) | 0.383 |
| **Recipient variables** |  |  |  |
| Male recipient, n (%) | 1,050 (73.5) | 451 (73.7) | 0.963 |
| Recipient age (years, M [P25, P75]) | 36.00 [30.00, 45.00] | 37.00 [30.00, 45.00] | 0.865 |
| HLA mismatch (numbers, M [P25, P75]) | 2.00 [1.00, 2.00] | 2.00 [1.00, 3.00] | 0.083 |
| Recipient BMI (kg/m^2^, M [P25, P75]) | 21.34 [19.25, 23.72] | 21.34 [19.27, 23.54] | 0.995 |
| Positive PRA, n (%) | 95 (6.6) | 48 (7.8) | 0.382 |
| **HMP parameters** |  |  |  |
| Initial flux (mL/min, M [P25, P75]) | 95.00 [80.00, 109.00] | 94.00 [78.00, 108.00] | 0.533 |
| Initial pressure (mmHg, M [P25, P75]) | 35.00 [30.00, 39.00] | 35.00 [30.00, 40.00] | 0.582 |
| Initial resistance (mmHg·mL^-2^·min^-1^, M [P25, P75]) | 0.35 [0.29, 0.42] | 0.34 [0.29, 0.41] | 0.632 |
| Terminal flux (mL/min, M [P25, P75]) | 110.00 [98.00, 121.00] | 110.00 [98.00, 120.00] | 0.495 |
| Terminal pressure (mmHg, M [P25, P75]) | 30.00 [30.00, 35.00] | 30.00 [30.00, 35.00] | 0.375 |
| Terminal resistance (mmHg·mL^-2^·min^-1^, M [P25, P75]) | 0.24 [0.20, 0.30] | 0.24 [0.20, 0.30] | 0.755 |
| Perfusion time (hours, M [P25, P75]) | 8.00 [5.00, 10.00] | 8.00 [5.95, 10.00] | 0.265 |

**Table S5.** Variables selected for predicting DGF in the model including ALT.

| **Variables** | **β** | **OR** | ***P-*value** | **VIF** |
| --- | --- | --- | --- | --- |
| Intercept | -7.36 | 0.00 | <0.001 | / |
| History of hypertension (Yes/No) | 0.89 | 2.43 | <0.001 | 1.09 |
| ALT (U/L) | 0.10 | 1.11 | 0.291 | 2.92 |
| AST (U/L) | 0.12 | 1.13 | 0.248 | 3.01 |
| Scr (μmol/L) | 0.49 | 1.63 | <0.001 | 1.47 |
| BUN (mmol/L) | 0.49 | 1.63 | <0.001 | 1.43 |
| Terminal flux (mL/min) | -0.02 | 0.98 | <0.001 | 1.20 |
| Terminal pressure (mmHg) | 0.03 | 1.03 | 0.041 | 1.23 |

VIF: variance inflation factor.

**Table S6.** Summary of biomarkers investigated in clinical machine perfusion studies.

| Biomarker | Sample number | Model | DOI | Year of Publication | Type of MP |
| --- | --- | --- | --- | --- | --- |
| Kidneys release (pro)renin, EPO, active vitamin D, and urodilatin | 28 | No | 10.1111/ctr.70163 | 2025 | NMP |
| ASAT, LDH, TIMP-2, and FABP | 25 | No | 10.1097/TP.0000000000005440 | 2025 | HMP and NMP |
| NGAL, LDH, GST-Pi, L-FABP, and IL-18 | 36 | No | 10.1038/s41467-025-59829-5 | 2025 | NMP |
| sDNA | 52 | No | 10.1097/TXD.00000000000001768 | 2025 | HMP |
| NGAL and LDH | 31 | No | 10.1590/S1677-5538.IBJU.2024.0166 | 2024 | HMP |
| TRL | 42 | No | 10.1097/TP.0000000000000004936 | 2024 | HMP and NMP |
| FMN | 422 | No | 10.1371/journal.pone.0287713 | 2023 | HMPO2 and HMP |
| Metabolites | 38 | No | 10.1097/TP.0000000000000004129 | 2022 | HMP |
| NGAL, KIM-1, L-FABP, cells, and cytokines | 12 | No | 10.3389/fmed.2021.801098 | 2022 | NMP |
| VCAM | 74 | No | 10.3389/fimmu.2022.966951 | 2022 | HMP |
| NAG and GST | 113 | Logistic, lack of set splitting | 10.1097/CM9.0000000000001867 | 2021 | HMP |
| Endothelins, IL-18, and NGAL | 22 | No | 10.3390/biomedicines9040417 | 2021 | HMP |
| ALCY, FABP5, IGHV2-26 and DSP | 44 | Logistic, lack of set splitting | 10.1111/tri.13984 | 2021 | HMP |
| GSH, GPX, CAT, SOD, GR, GST, TBARS, and MDA | 26 | No | 10.3390/antiox10081263 | 2021 | HMP |
| FMN | 11 | No | 10.1097/TXD.000000000000001046 | 2020 | NMP |
| Cytotoxic extracellular histones | 390 | No | 10.1097/TP.00000000000001590 | 2017 | HMP |
| KIM-1, IL-18, and L-FABP | 671 | No | 10.1111/ajt.13655 | 2016 | HMP |
| miR-21 | 11 | No | 10.1111/ctr.12679 | 2016 | HMP |
| Alpha and pi-GST | 428 | No | 10.1111/ajt.12635 | 2014 | HMP |
| GST, LDH, H-FABP, redox-active iron, IL-18, and NGAL | 335 | Logistic, lack of set splitting | 10.1097/TP.0b013e31827908e6 | 2013 | HMP |
| TNF-α, IL-2, IL-1β, and sICAM-1 | 104 | No | 10.1016/S1743-9191(13)60029-1 | 2013 | HMP |
| GST, α-GST, and lipid peroxidatio | 111 | No | 10.1016/j.jss.2012.04.071 | 2013 | HMP |
| GST, NAG, and H-FABP | 306 | No | 10.1097/TP.0b013e3181f5c40c | 2010 | HMP |
| Ala-AP, FABP, and GST | 69 | No | 10.1258/000456303321610565 | 2003 | HMP |

MP: machine perfusion; NMP: normothermic machine perfusion; HMPO2: oxygenated hypothermic machine perfusion; EPO: erythropoietin; ASAT: aspartate aminotransferase; LDH: lactate dehydrogenase; TIMP-2: tissue inhibitor of metalloproteinases-2; FABP: fatty acid-binding protein; NGAL: neutrophil gelatinase-associated lipocalin; GST: glutathione s-transferase; IL-18: interleukin-18; sDNA: soluble DNA; TRL: tissue-resident lymphocyte; FMN: flavin mononucleotide; KIM-1: kidney injury molecule-1; VCAM: vascular cell adhesion molecule; NAG: N-acetyl-β-D-glycosaminidase; ALCY: ATP‐citrate synthase; FABP5: fatty acid–binding protein 5; IGHV2-26: immunoglobulin heavy variable 2-26; DSP: desmoplakin; GSH: glutathione; GPX: glutathione peroxidase; CAT: catalase; SOD: superoxide dismutase; GR: glutathione reductase; TBARS: thiobarbituric acid reactive substances; MDA: malondialdehyde; miR-21: microRNA-21; TNF-α: tumor necrosis factor-alpha; IL-2: interleukin-2; IL-1β: interleukin-1 beta; sICAM-1: soluble intercellular adhesion molecule-1; Ala-AP: alanine aminopeptidase.

**Figure S1.** The comparison of Scr in recipients at one, six, and twelve months after kidney transplantation according to thresholds of initial resistance (A, D, G, thresholds: 0.35 mmHg·mL^−2^·min^−1^), terminal resistance (B, E, H, thresholds: 0.24 mmHg·mL^−2^·min^−1^) and terminal flux (C, F, I, thresholds: 112.18 mL/min). Scr: serum creatinine; HMP: Hypothermic machine perfusion.

**Figure S2.** Pearson correlation analysis between serum ALT and AST levels in the train set. Each point represents an individual sample. ALT: alanine aminotransferase; AST: aspartate aminotransferase. R denotes the Pearson correlation coefficient, indicating the strength and direction of the linear relationship between ALT and AST.

**Figure S3.** ROC curves for model performance in the validation cohort. The ROC curves illustrate the discriminative performance of the full multivariable model and two reduced models only including Scr or BUN, respectively. ROC, receiver operating characteristic; Scr, serum creatinine; BUN, blood urea nitrogen.

**Figure S4.** QR code for accessing the web-based DGF prediction tool. The QR code was generated from the web version of the predictive model (https://boqingdong.shinyapps.io/XJTU_KT_DXM__DGF/). By scanning the code, users can directly access the online tool to estimate the risk of delayed graft function after kidney transplantation.
